# Why medical students do not choose a career in geriatrics: a systematic review

**DOI:** 10.1186/s12909-015-0384-4

**Published:** 2015-06-05

**Authors:** Ariadne A. Meiboom, Henk de Vries, Cees M.P.M. Hertogh, Fedde Scheele

**Affiliations:** 1 Department of General Practice & Elderly Care Medicine, VU University Medical Center, Van der Boechorststraat 7, 1081 BT Amsterdam, The Netherlands; 2 Department of Research in Education, VU University Medical Center, Van der Boechorststraat 7, 1081 BT Amsterdam, The Netherlands

**Keywords:** Medical students, Career choice, Geriatrics

## Abstract

**Background:**

While the demand for doctors specialised in the medical care of elderly patients is increasing, the interest among medical students for a career in geriatrics is lagging behind.

**Methods:**

To get an overview of the different factors reported in the literature that affect the (low) interest among medical students for a career in geriatrics, a systematic literature search was conducted using PubMed, Embase, PsycINFO, and ERIC. Quality assessment criteria were applied.

**Results:**

Twenty studies met the criteria and were included in the review.

In relation to the *nature of the work*, the preference of medical students is young patients, and acute somatic diseases that can be cured. The complexity of the geriatric patient deters students from choosing this specialty. *Exposure* by means of pre-clinical and particularly clinical education increases interest. The lack of *status* and the *financial* aspects have a negative influence on interest.

**Conclusion:**

Exposure to geriatrics by means of education is necessary. The challenge in geriatric education is to show the rewarding aspects of the specialty.

**Electronic supplementary material:**

The online version of this article (doi:10.1186/s12909-015-0384-4) contains supplementary material, which is available to authorized users.

## Background

Healthcare has to be arranged for the increasing proportion of older people in the population. According to the United Nations the number of people over 80 is likely to quadruple in most countries, reaching 400 million by 2050 [1].

One solution to meet the health care needs of the growing number of elderly is to increase the number of doctors specialized in the medical care of elderly people. Despite differences between countries, the main problem is that the interest among medical students in a career in geriatrics is lagging behind. In the United States, for example, 44 % of the geriatric medicine first-year fellowship training slots were left unfilled in 2008, while many more geriatricians are needed in the future [2].

Many expert views can be found in the literature regarding the issue of insufficient interest in geriatrics. The main themes that are highlighted are (lack of) exposure to the field, finances and status, and the nature of the work.

Regarding exposure, both a lack of exposure to geriatrics and the scarcity of positive role models are said to contribute to the students’ lack of interest in geriatrics [3, 4]. In addition, the fact that there are not enough faculty members to teach, the lack of visibility of research programmes in comparison with other specialties, and a scarcity of trained leaders in geriatrics are also said to play a role [3, 4].

The relatively low financial rewards (at least in some countries) and low status associated with the field are indicated as contributing factors to a lack of interest in geriatrics [3–5].

In relation to the nature of the work, treating a high number of elderly patients with chronic illnesses is reported to be less attractive than curing younger patients with acute illnesses [5].

However, the experts do not present the evidence for the factors they mention. Nor do they offer other possible explanations for the limited interest in geriatrics.

The above brings us to the following question:

What is known from scientific research regarding the factors that contribute to the interest or lack of interest of medical students in a career in geriatrics?

We performed a systematic search of the literature to get an overview of the different factors that are relevant in the decision of medical students to choose a career in geriatric medicine.

## Methods

A literature search was conducted by the researcher and a senior librarian on February 8, 2013, using PubMed, Embase, ERIC and PsycINFO. The search (Mesh) terms used were geriatrics, career, career mobility, career planning, career choice, specialisation, medical student, and clinical education.

Only original research was selected. A publication was deemed relevant if it included medical students’ interest in geriatrics in relation to possible influencing factors. No restriction was made as to the year of publication. Only studies written in English, French, German or Dutch were selected. There were no restrictions regarding country of origin.

Titles, abstracts and if necessary full text articles were independently reviewed for relevance by two researchers (AM, HdV). Disagreements were resolved through discussion with a third reviewer (FS).

Reference lists were checked for additional publications, which in turn were checked for relevance using the same criteria.

### Quality assessment

The quality of each quantitative study was assessed independently by two researchers (AM, HdV) on the basis of the validity scale developed by Bland et al., which provides a systematic approach for a “non-statistical meta-analysis” of the literature [6]. The following data were extracted, using a predetermined form: author, year, type of study, data source, theory or model based, number of students as subjects, number of schools as subjects, sample size, level of training of respondents, variables. Quality scores ranged from 0 (minimum) to 100 (maximum). A higher quality score was given for good internal validity, the use of reliable measuring instruments, the research being based on a theory, and larger samples and higher response rates in the research. If the two assessors could not reach consensus, the decision was made by a third researcher (FS).

Studies with a cumulative rating of 45, the cut-off chosen by Bland, were considered to have a sufficiently high level of evidence, and were included.

For each qualitative study, it was ascertained whether it contained a good description of the research team and reflexivity, the study design and the analysis and findings, the three domains of the consolidated criteria for reporting qualitative studies (COREQ) checklist [7]. Detailed description of at least two of these items meant inclusion of the study.

### Specialty choice model

To organise our data we used the model of medical students’ specialty choice developed by Bland and Meurer [8]. This model is based on the premise that medical students will try to match the perceived characteristics of the specialty to their career needs, based on their values which in turn are based on student characteristics like personality, preschool experiences, and demographic factors. Furthermore student values and the way students perceive specialty characteristics are influenced by medical school characteristics such as values and culture of the institution, faculty composition and curriculum.

To summarize the literature we used the main components from the Bland-Meurer model:

‘medical school characteristics’ (a combination of type of school, mission and structure, faculty composition, admission committee, faculty values, curriculum committee, student composition, institutional culture and curriculum), ‘medical student characteristics’, (a combination of medical students’ incoming values, graduate values and needs to satisfy) and ‘medical students’ perception of characteristics of geriatrics’.

To determine whether information is available for each component of the model, the results are summarized in relation to this model.

### Ethical approval

Ethical approval is not necessary in case of this literature review.

## Results

### Articles

The literature search yielded 326 citations from PubMed. The search added 240 citations in Embase, 59 in ERIC and 22 in PsycINFO.

Many publications concerned the evaluation of educational programmes and/or the attitude of the medical student in relation to the elderly or the elderly patient. They were only selected if they also measured the medical student’s interest in geriatrics.

**Fig. 1:**
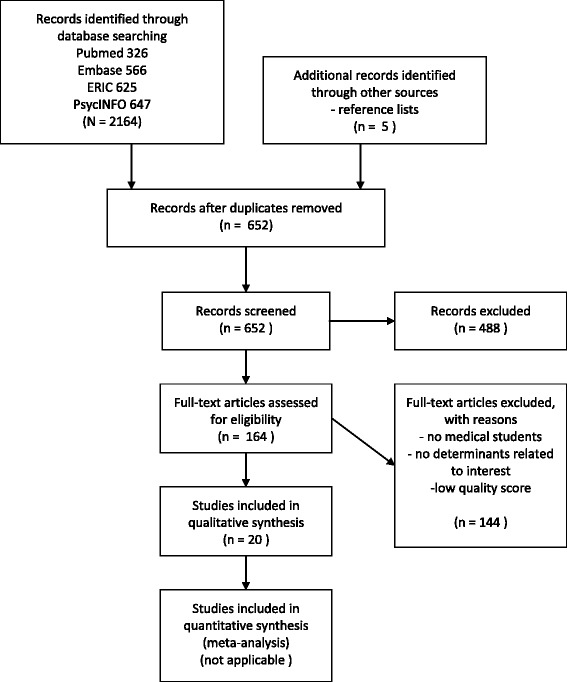
Search history

On the basis of the inclusion criteria, 281 titles were selected. Abstracts were available for 174 titles, of which 122 were excluded. From the 159 requested full articles, 18 were found to be relevant. In addition, five relevant references were selected, resulting in a total of 23 selected articles, i.e. three qualitative studies and 20 quantitative studies. Of the quantitative studies, two were excluded on the basis of a quality score of less than 45. One of the qualitative studies was excluded on the basis of insufficient quality (Fig. 1). An overview of the included articles, including the assigned quality scores, can be found in Additional file 1.

Factors (see Table 1):

**Table 1:** Factors Examined as Potentially Influencing an Interest in a Career in Geriatrics by medical students

Factors	Influence to the interest in geriatrics	
	*Articles qualitative*	*Articles quantitative*
*Medical school characteristics*		
Department of geriatric medicine		9 + s
Preclinical education in geriatrics		10 + ns, 11 + ns, 12 ns
Preclinical elective education in geriatrics		14 + s
Clinical education in geriatrics		14 + s, 16 + s, 17+, 18+, 19 ns
Knowledge of geriatrics		12 ns, 22 ns, 23 ns, 24 ns
*Medical student characteristics*		
Previous personal contact with elderly/care of elderly	25+	23 ns, 19 + s, 22 + s, 15 ns, 20 ns, 23 ns, 26 ns, 27 + s
Female gender		15 ns, 20+, 22+, 26+
Age of the student		15 ns, 20 ns, 26 ns
Ethnic backgrounds of the student		15 ns, 26 ns
Medical students’ fear about aging and death	25+	
Attitudes towards the elderly		15 + s, 22 +s, 23 ns, 26 + s
Seeing themselves as a healer	25-	
Seeing themselves as a caretaker	25+	
*Medical students’ perception of characteristics geriatrics*		
Few are cured/no direct benefit	25-, 28-	
Management of chronic illness		20-s, 17-s
Focus on quality of life	25 − +	20-s
Complexity of geriatric patients	28-(+)	
Not feeling comfortable with ambiguity		20-s
Low financial reward	28-	20-s, 17-s
Possibility to work part-time/perceived lighter call schedule		17 + s
Length of training (five years)		20-s
Lack of prestige	28-	20-s

Numbers are reference numbers

*+s* significant (*P* < .05) positive influence, *+* positive influence, significance not mentioned, *ns* not significant (*P* > .05)*, -s* significant negative influence, *-*negative influence, significance not mentioned, (*+*) positive influence for a small percentage of students

The findings from the 20 studies are categorised according to the summarized components of the Bland-Meurer model.

In each category the qualitative studies are described first, followed by the quantitative studies.

#### Medical school characteristics

### Faculty members and role models

In the UK, in a medical school with a Department of Health Care of the Elderly, significantly more students appeared to have an interest in geriatrics than in a medical school without a Department of Health Care of the Elderly [9].

### Pre-clinical education

Several studies looked at the influence of geriatric courses in the first year of medical school on medical students’ interest in geriatrics as a specialty.

The courses varied from 25 h to six days in total.

In two studies a trend was observed of increasing interest for geriatrics as a possible specialty choice as compared to before these courses [10, 11].

One study found no difference in interest before versus after education [12].

One medical school implemented the Geriatrics Continuity of Care Track, consisting of six sessions with three components; a 1-h didactic presentation; a visit to an assigned older volunteer; responses to web-based reflection questions. No significant increase in interest in geriatrics was found after participation in the programme [11].

After a one-week module on aging, consisting of sessions on topics ranging from molecular biology to societal aspects of aging, an increase was shown in the choice for geriatrics as a specialty. However, this increase was not statistical significant [12]. No difference in interest in working with the elderly was found before versus after a 24-h course with lectures, discussions and interviews of elderly people [13].

A study that looked into the influence of different didactic methods did not show a measurable difference in interest in geriatrics between a 3-h didactic session and a 3-h experiential learning session [13].

One medical school implemented a voluntary extracurricular programme in which first-year medical students were partnered with elderly who were active and living independently [14]. Throughout the school year the students and their senior partners met several times. Prior interest in geriatrics and participation in the programme were significant indicators for interest in a career as a geriatrician.

### Clinical education

Five studies looked into the interest in geriatrics after clinical education.

A statistically significant increase was seen after a clinical training programme in geriatric medicine lasting eight working days [15, 16]. In one medical school students were randomly allocated to a geriatric medicine attachment or a general medicine attachment. Afterwards, significantly more of the students attached to geriatric medicine were prepared to consider a career with elderly patients [15, 16]. Two other studies also reported an increase in interest after an attachment to healthcare of the elderly, lasting five weeks and one month respectively, but the statistical significance is not mentioned [17, 18].

No difference in interest was observed between students who undertook a four-week geriatric rotation within a primary care clerkship of eight weeks, and those who did not [19].

### Longitudinal effect of education

Only three studies examined the interest in geriatrics in a longitudinal way. Interest decreased significantly one year after preclinical education in geriatrics as compared to the level immediately after the education [13, 20]. Also, the interest in geriatric medicine as a career showed a significant decrease between the completion of a fourth-year health care of the elderly attachment and graduation [21].

### Knowledge

Knowledge of geriatrics per se did not appear to be a significant factor [12, 22–24].

#### Medical student characteristics

##### Pre-medical school experiences

Qualitative research has shown that little or negatively tainted previous experience with elderly persons had a negative influence on interest [25].

Quantitative research findings in this area vary.

Previous personal contact with elderly persons did not have any influence in one study [23]. In another study the degree of positive feelings about previous contact with the elderly was significantly and positively related to intentions to work with the elderly [19]. Previous experience with elderly care had a positive influence on interest in geriatrics in one study [22]. In four other studies it appeared to have no influence [15, 20, 23, 26].

The proportion of medical students reporting a positive work or volunteer experience with seniors was found to be significantly higher among those interested in geriatric medicine than among those not interested, indicating that the quality of the previous experience is more important than the quantity [27].

### Demographic factors

Different studies have looked at a number of demographic factors. More women than men were interested in geriatrics [20, 22, 26]. Another study found no difference between male and female students [15]. Different ethnic groups of medical students in the United States and Singapore showed no difference in interest [15, 26]. Also, no difference was found for different age groups [15, 20, 26].

However, more men appeared to have a significant preference for younger patients, acute illnesses, diagnostic procedures, and cognitively intact elderly patients [13]*.*

### Fears and goals

Qualitative research reported that students interested in geriatrics expressed more fear about growing old - for themselves and others - and about death, especially the death of others close to them, but also the death of their patient [25].

The authors hypothesized that these fears drive the students to help others in the areas that they fear. In the same study students who did not have an interest in geriatrics saw medicine as fast and exciting, with the goal of adding many years to the life of the patient, resulting in the belief that working with an older patient is not rewarding. Students with a moderate to strong interest in geriatrics on the other hand, saw themselves more as care-takers, looked forward to comforting patients and believed that raising the quality of life of a patient can also be rewarding.

### Attitude

A few studies looked at the relationship between the attitudes of medical students towards the elderly person or to the medical care for the elderly patient and their willingness to consider a career in geriatrics.

In three studies a significant relationship was observed between interest in geriatrics as a career choice and their attitude [15, 22, 26]. However, another study did not find such a relationship [23].

#### Medical students’ perception of characteristics of geriatrics

### Chronicity of disease

Qualitative research has shown that students are discouraged because it is impossible to see the direct effects of treatment or curation [25, 28]. In addition, many of them see not being able to solve all of their patients’ problems as a personal failure [28]. The students experience the decline and death of their patients as depressing [25, 28].

Quantitative research has shown that students not interested in geriatrics perceived the chronicity of disease and the focus on quality of life as a barrier to geriatric medicine as a career choice [20]. Long-term care is found less attractive by medical students not interested in a career in geriatric medicine [27].

### Complexity of geriatric patients

Students find the geriatric patients too complex, according to qualitative research. They feel overwhelmed by the poor health, the number of medical problems, atypical disease presentation, and polypharmacy [28]. To assess and manage a geriatric patient takes too much time in their opinion [28].

### Not feeling comfortable with ambiguity

Quantitative research has highlighted that ‘not feeling comfortable with ambiguity’ in relation to the complexity of patients was a bigger barrier to selecting geriatric medicine as a career for students who are not interested in geriatrics than for those who are [20].

### Financial and lifestyle considerations

The low financial reward as a negative influencing factor is reported in both qualitative and quantitative research [20, 27, 28].

The possibility of working part-time and the perceived lighter call schedule was found to be of importance to students in the clinical phase who had some interest in geriatrics [27].

The length of training (five years) was a barrier for first-year students in Canada to selecting geriatric medicine as a career [20].

### Status

Quantitative research has shown that for students not interested in geriatrics, prestige was a deterrent, although not a major deterrent [20].

### Fears

According to qualitative research medical students expect to have difficulty with the ethical dilemmas and are afraid of unrealistic expectations on the part of the families of patients [28]. Students not interested in geriatrics feared their patients being non-compliant and thought this would be frustrating [25].

### Patients’ age

In addition, students not interested in geriatrics rated caring for younger patients as an important practice characteristic [20].

### Positive factors

On the other hand, research has also revealed characteristics of geriatrics that positively influence an interest in this field among a small proportion of students:

In qualitative studies, some students found the geriatric patient a challenge and end-of-life care rewarding [25, 28].

Quantitative research has described that the focus on the entire patient as opposed to organ systems, the intellectual challenge and opportunities for research as well as the experience of perceived rewards in the field of geriatrics are a positive influence in a small proportion of students [19, 27].

## Discussion

We conducted a literature review to get an overview of the different factors that contribute to the interest or lack of interest in a career in geriatrics among medical students. The following aspects are worth mentioning.

### Medical school characteristics

An increase in interest in geriatrics was seen particularly after clinical education in geriatrics. But the question is how to maintain this increased interest throughout the following years in medical school.

A visible department of health care of the elderly did have a positive influence on the interest in geriatrics among medical students.

Bland and Meurer distinguished more medical school components in their model, as mentioned in the methods section, e.g. mission and structure, institutional culture and faculty values. We did not find any geriatrics-oriented research on these components.

### Medical student characteristics

The findings regarding demographic factors vary considerably.

A positive correlation was found between an interest in geriatrics and attitude (towards the elderly person or the medical care for the elderly patient) in three of the four studies. However, these studies utilised the UCLA Geriatric Attitude Scale, a scale validated for primary care residents, not for medical students [29]. In addition, one of the studies also used the Maxwell-Sullivan Attitude Scale, a commonly used but non-validated scale designed for family practice residents [30, 31]. The items of both scales contain a mix of attitude in relation to the elderly patient and elderly medical care.

The possibility of working part-time and the perceived lighter call schedule are seen as attractive factors. This may provide an opportunity to draw more students towards geriatrics.

### Characteristics or perceived characteristics of geriatrics

Geriatrics has a number of characteristics that the majority of students do not find attractive. These involve working with chronically ill patients, working in long-term care, less curable diseases and dealing with only elderly patients. It is also becoming clear that the complexity of the geriatric patient deters students. However, these students were not exposed to geriatric patients in a geriatric clerkship, only in other clerkships. In other words, they lacked the potentially positive experience of learning to manage these complex patients. A study on the perceived needs in geriatric education also described that medical students and residents found caring for the elderly unattractive, because they do not know how to deal with the complexity involved, multimorbidity, shorter life expectancy, and balancing treatment of diseases with quality-of-life and psychosocial issues. However, when they learned to manage these patients, it was experienced as ‘rewarding’ [32]. So, education and learning can transform an overwhelming experience into a rewarding experience.

The low financial rewards and the lack of prestige of the specialty do have a negative impact on the specialty choice for geriatrics. However, the impact of the low prestige of the field seems to be small. According to Album the prestige of specialties, the prestige of diseases, and the characteristics of the patients having the disease are interrelated [33]. Specialties that are considered a less biomedical type of medicine were seen as less prestigious. Furthermore, specialties with a low level of prestige are associated with, among other things, elderly patients, chronic conditions, and less visible treatment procedures [33, 34].

A study on specialty choices by medical students in general reveals that “type of patient problems encountered” was the factor rated as the most influential [35].

The Theory of Reasoned Action (TRA) proposes that the best predictor of behaviour, like specialty choice, is the intention to perform that behaviour [36]. Intentions consist of attitudes and subjective norms. In this case this means attitudes regarding geriatrics or the medical care for elderly patients. Subjective norms, e.g. the importance of the opinions of others, may consist of disregard for geriatrics in their environment.

Based on the TRA and the finding that “type of patient problems encountered” was rated as the most influential factor of specialty choice in general, it seems worthwhile to improve medical students’ perception of and attitudes towards the type of patient problems in geriatrics.

#### Strengths/weaknesses

A strength of this study is that it wants to systematically map out the factors that play a role in medical students choosing geriatrics, which has not been done before. Unfortunately, the research in this area is insufficiently consistent for statistical meta-analyses. For instance, there is no uniform definition of students’ interest in geriatrics. In addition, most studies look at only a few variables, and the underlying factors are defined and also measured in different ways, so that quantitative comparison is impossible. In some studies it was not possible to test the significance of the different factors due to the small number of students with an interest in geriatrics as a career. Besides, not all potentially relevant factors have been studied thus far, e.g. faculty culture and values.

In short: the current state of the literature does not allow for a complete understanding of the underlying factors of medical students’ interest in geriatrics. Also we could not determine from the literature if medical students with an interest in geriatrics actually choose this speciality.

Another strong point is that we assessed the quality of each article using the Bland validity score, and this article shows the validity of results for each study. Besides, the studies that were not included on the basis of an inadequate quality score did not show a different picture. The use of the Bland-Meurer model was helpful in structuring and understanding the data.

A limitation may be found in the generalizability of the findings, since the studies were conducted in different countries and different decades. However, factors found in older studies also emerge in more recent ones. Only the factor ‘a visible department’ is found only once in an article from 1986. However, this factor was not included in the other studies, and we expect it will have the same effect today”.

#### Recommendations

### Policy

Exposure to geriatrics by means of a visible department and faculty members, pre-clinical and particularly clinical education, appears to be important.

The challenge for politics and professional associations is to improve the image of geriatric medicine as well as the financial rewards. Prestige and high reimbursement are interrelated and associated with diagnostic and therapeutic technical procedures. The time has come to realize a more appropriate reimbursement for managing patients with multiple chronic conditions.

### Medical education

To increase the level of enthusiasm of medical students for geriatrics, it is important to show them the rewarding aspects of long-term care. Also, they need guidance to learn how to manage the complexity of geriatric patients, as well as deal with ethical dilemmas.

A mandatory geriatrics clerkship or clerkship in a nursing home can show students the challenge of solving complex clinical puzzles. To learn to manage the complexity of geriatric patients the students have to learn to move from a disease-oriented approach to a patient goal-oriented approach.

Intolerance of uncertainty is associated with reliance on high technology and is a predictor of a non-primary care specialty choice [37, 38]. As medical students with a poor tolerance of uncertainty consider the work of a general practitioner to be too challenging, we may assume that they are also put off by the complexity of the geriatric patient [37, 38]. The learning environment of a nursing home, with its paucity of advanced diagnostic tools, gives medical students the opportunity to develop their clinical and deliberate decision-making skills instead of relying on the more standardized treatment options in the hospital [39].

Besides, to increase the number of geriatricians, tolerance of uncertainty should be a criterion for admission to medical school.

In addition to increasing the number of geriatricians, we have to ensure that all future physicians have the skills to manage the problems of elderly patients, as they will be spending more and more time caring for these patients. Hence, medical curricula as well as residency training programs should pay more attention to the principles of geriatrics. The two following examples illustrate how this can be realized, for medical students and for chief residents.

In 2007 the AAMC Geriatric Competencies for Medical Students were developed through a multi-method consensus process [40, 41]. This document consists of a minimum set of 26 geriatrics-specific competencies to be implemented in medical schools in the United States to prepare graduating medical students for the tasks they have to perform as a first-year resident in caring for geriatric patients.

The ADGAP Chief Resident Immersion Training (CRIT) in the Care of Older Adults consists of a two-day program for chief residents in medical or surgical residency training and provides case-based training in geriatric principles with a focus on the care of complex older patients [42].

### Research

Both explanatory and longitudinal research is needed to gain more insight into the weight and the relationship of the different factors over time, so that interventions can be more focused.

It seems worthwhile to examine whether the student perception of the characteristics of geriatrics is influenced by a clerkship in geriatrics and whether this perception is in line with the actual characteristics of this field. It is also interesting to ascertain whether the potential change in perception affects the interest of students.

In addition, it is important to see which forms of education have more effect on student perception and interest and whether the length of the clerkship plays a role in this.

As the complexity of the geriatric patient appears to be an important negative factor. It is interesting to see which forms of education may improve the sense of competence and whether or not this influences interest.

This study focuses on medical education. However, a number of physicians will make their career choice during postgraduate clinical training. Therefore, it is also relevant to search for interventions that are useful in postgraduate clinical training.

## Conclusion

In relation to the lack of interest in a career in geriatrics among medical students, evidence was found for the following themes arising from expert views: a. lack of exposure, b. low status and low financial reward, c. nature of the work. Regarding the latter, supplementing what expert views say, it is becoming clear that students are deterred by the complexity of the geriatric patient.

This review presents an overview of the currently known factors that may underlie the limited interest of medical students in geriatrics.

To guarantee strong medical care for frail, elderly people in our ageing society, it is important to address these factors now.

## Additional file

Additional file 1:
**Summary of publications about interest in geriatrics by medical students.**

## Abbreviations

UCLA GAS: University of California Los Angeles Geriatric Attitude Scale
MSAS: Maxwell-Sullivan Attitude Scale
UK: United Kingdom
