# Supplementary material for: Why medical students do not choose a career in geriatrics: a systematic review
Source: BMC Med Educ. 2015 Jun 5;15:101. doi: 10.1186/s12909-015-0384-4 (PMC4470031; doi:10.1186/s12909-015-0384-4)
Supplement: Additional file 1: — Summary of publications about interest in geriatrics by medical students. [file 12909_2015_384_MOESM1_ESM.pdf]

Additional file 1. Summary of Publications about Interest in Geriatrics by Medical Students.

Article	*Author*	*Year*	*Population, medical students and response rate (rr)*	*Design*	*Variables (Independent Variables Dependent Variables)*	*Measurement tool*	*Quality score*
9	Wattis et al.	1986	rr 94% ® 81 rr, 77% ® 117, clinical students before attachment	Etiologic-observational	IV: medical school<br>DV: interest	attitude: modified ASD Rosencranz-McNevin and a Likert scale with specific medical content	70
10	Alford et al.	2001	rr 82% ® 404 first-year students	Etiologic-observational	IV: education<br>DV: interest and attitude	attitude: own questionnaire, some validity and reliability	66
11	Eskildsen et al.	2009	rr 99% ® 129, pre-clinical students	Pre-experimental	IV: education<br>DV: interest and attitude	attitude: UCLA-GAS	60
12	Carmel et al.	1992	rr 86% ® 127 first-year students	Etiologic-observational	IV: education<br>DV: knowledge, attitude, work preference	attitude: own list	59
13	Diachun et al.	2006	rr 42% ® 42 first-year students,	Etiologic observational	IV: different form of education<br>DV: interest and attitude	attitude: Palmore	67
14	Lu et al.	2010	rr 71% ® 147 first-year students	Etiologic-observational	IV: voluntary extra-curricular education<br>DV: interest and prior interest	attitude: ASD Rosencranz-McNevin	74

Article	*Author*	*Year*	*Population, medical students and response rate (rr)*	*Design*	*Variables (Independent Variables Dependent Variables)*	*Measurement tool*	*Quality score*
15	Hughes et al.	2008	rr 96% ® 163 first-year students, rr 58 % ® 70 fourth-year students	Pre-experimental	IV: attitude. DV: interest<br>IV: clinical education DV: interest and attitude	UCLA GAS	53
16	Peach et al.	1982	rr 71% ® 129, clinical phase	Pre-experimental	IV: education<br>DV: interest and attitude	Own questionnaire, some validity and reliability	62
17	Sainsbury et al.	1992	rr not known. 68 in total, clinical phase	Pre-experimental	IV: education<br>DV: attitude towards elderly, medical attitude towards elderly, interest	Rosencranz-McNevin Likertsale (attitude elderly medical care)	60
18	Smith et al.	1989	rr 99% ® 122 , clinical phase	Observational	IV: education DV: attitude regarding elderly and attitude regarding elderly medical care	attitude: ASD Rosencranz-McNevin	50

Article	*Author*	*Year*	*Population, medical students and response rate (rr)*	*Design*	*Variables (Independent Variables Dependent Variables)*	*Measurement tool*	*Quality score*
19	Green et al.	1983	rr 100% ® 148 third-year students during general practice clerkship	Experimental	IV: Geriatric rotation<br>DV: Intention to work with the elderly	Multidimensional questionnaire, compiled from previously developed and tested instruments or by researchers on the basis of face validity. Factor analysis performed.	80
20	Diachun et al.	2006	rr 90% ® 108 first-year students,<br>follow up in the second year, rr of 80% ® 96 students	Observational	IV: factors<br>DV: interest	No known measurement tool. Measured validity and reliability themselves.	59
21	Sainsbury et al.	1994	rr 61,8% ® 102 graduates	Etiologic-observational	IV: going through entire clinical phase/other clerkships<br>DV: attitude elderly medical care	Likert-scale, used before.	66

Article	*Author*	*Year*	*Population, medical students and response rate (rr)*	*Design*	*Variables (Independent Variables Dependent Variables)*	*Measurement tool*	*Quality score*
22	Fitzgerald et al.	2003	rr 98% ® 171 first-year students	Observational	IV: knowledge, attitude, previous experience with elderly care<br>DV: interest	UCLA GASMSAS	54
23	Perrotta et al.	1981	rr 100% ® 127 first-year students	Observational	IV: knowledge and attitude regarding elderly. DV: attitude GM of geriatric patient	Kogan (attitude elderly). Attitude GM: developed by Gale and Livesly ad Cichetti and colleagues. Knowledge: Palmore.	55
24	Michielutte et al.	1985	rr 92% ® 403 students	Observational	IV: positive experience elderly care, intention internal medicine or general practice<br>DV: interest	Palmores Facts on Aging Quiz (knowledge and perception) (25)	59
25	Schigelone et al.	2004	20 first-year students	Qualitative	Experience with elderly, beliefs about geriatrics, beliefs about medicine	Interviews	
26	Chua et al.	2008	rr 97.6% ® 244 first-year students	Observational	IV: gender and attitude<br>DV: interest	UCLA GAS	45

Article	*Author*	*Year*	*Population, medical students and response rate (rr)*	*Design*	*Variables (Independent Variables Dependent Variables)*	*Measurement tool*	*Quality score*
27	Torrible et al.	2006	rr 45% ® 140 senior	Observational	IV: factors DV: attractiveness of GM	GRIST survey	47
28	Bagri	2010	30 fourth-year students	Qualitative	Factors	Focus groups	
